# Effects of Short-Term Exposure of Chloramine-T Solution on the Characteristics of Light-Cured and Chemical-Cured Adhesives

**DOI:** 10.3390/polym15193995

**Published:** 2023-10-05

**Authors:** Yunqing Liu, Norihito Sakaguchi, Masahiro Iijima, Md Refat Readul Islam, Jiayuan Zhang, Rafiqul Islam, Monica Yamauti, Hidehiko Sano, Atsushi Tomokiyo

**Affiliations:** 1 Department of Restorative Dentistry, Faculty of Dental Medicine, Hokkaido University, Sapporo 0608586, Japan; rony12cdc@den.hokudai.ac.jp (R.I.); myamauti@den.hokudai.ac.jp (M.Y.); sano@den.hokudai.ac.jp (H.S.); tomokiyo@den.hokudai.ac.jp (A.T.); 2 Center for Advanced Research of Energy Technology, Faculty of Engineering, Hokkaido University, Sapporo 0608628, Japan; sakaguchi@eng.hokudai.ac.jp; 3 Department of Oral Growth and Development, Division of Orthodontics and Dentofacial Orthopedics, Health Sciences University of Hokkaido, Ishikari-Tobetsu 0610293, Japan; iijima@hoku-iryo-u.ac.jp; 4 Department of Restorative Dentistry, Graduate School of Dental Medicine, Hokkaido University, Sapporo 0608586, Japan; refatrislam@den.hokudai.ac.jp (M.R.R.I.); zhangjiayuan@den.hokudai.ac.jp (J.Z.)

**Keywords:** microtensile bond strength, bonding performance, nanoleakage, FIB-SIM, mechanical properties, interfacial characteristics

## Abstract

This study evaluated the effect of a 0.5% chloramine T solution on a chemical-cured universal adhesive by comparing the light-cured, one-step, self-etch adhesive for the bonding performance, mechanical properties, and resin–dentin interfacial characteristics. Caries-free human molars were randomly assigned into eight groups based on the bonding systems employed (Bond Force II, BF and Bondmer Lightless, BL), the immersion solutions used before bonding (0.5% chloramine T solution and distilled water), and the immersion durations (5 and 60 min). Microtensile bond strength (μTBS), nanoleakage evaluation, and nanoindentation tests were performed, and the surface morphology of the resin–dentin interface was examined using a focus ion beam/scanning ion microscopy system. Immersion in chloramine-T for 5 min significantly decreased the μTBS of Bondmer Lightless (from 22.62 to 12.87 MPa) compared with that in distilled water. Moreover, there was also a decreasing trend after immersing in chloramine-T for 60 min (from 19.11 to 13.93 MPa). Chloramine T was found to have no effect on the hardness, elastic modulus, or morphological characteristics of the ion-beam milled resin–dentin interfacial surfaces in the tested adhesives, suggesting that chloramine T might reduce the bond strength by interfering with the interaction and the sealing between the adhesive resin and dentin in the chemical-cured universal adhesive, albeit without affecting the mechanical properties.

## 1. Introduction

“Dental adhesive” has created a new era in the field of dentistry due to its diverse use in the different branches of dentistry, including operative dentistry, endodontics, orthodontics, pediatric dentistry, and prosthetic dentistry [1,2]. In the field of restorative dentistry, clinicians use it frequently for direct restorative purposes where its use can be combined with resin composite that facilitates durable bonding to the tooth structure. However, dental adhesive is also very popular for its adhesion properties to be used in indirect restorations, such as an inlay, onlay, and a crown.

Based on the tooth adhesion strategies, dental adhesives can be divided into two main categories: “etch-and-rinse” (ER) and “self-etch” (SE) adhesives [3,4]. The ER bonding process involves using phosphoric acid to etch the tooth surface, which removes the smear layer entirely [5]. Also, ER ensures the micromechanical interlocking both in the enamel and dentin by promoting diffuse-based adhesion strategies. Nevertheless, ER has some drawbacks, like collagen exposure and the demineralization of dentin. Also, ER might increase the risk of micro or nano leakage and enzymatic biodegradation [4].

On the other hand, the SE bonding mode utilizes functional monomers such as 10-methacryloyloxydecyl dihydrogen phosphate (10-MDP or MDP), which eliminates the need for a separate etching step and subsequent rinsing with water [6,7,8,9]. However, SE bonding is generally less effective than ER bonding when it comes to enamel [10], as enamel is better treated with phosphoric acid in a separate and selective etching process.

In order to overcome those issues, most recently, a new generation of adhesives has been commercially marketed, known as universal adhesives (UAs) [11,12]. It offers the flexibility to clinicians to use in either the ER mode, the SE mode, or a combined approach where ER is applied to enamel and SE to dentin [13,14,15]. Typically, UAs can be one-bottle or two-bottle adhesives that contain the primer and adhesive resin, simplifying the clinical procedure and reducing the application time, claiming reductions in technical sensitivity [9,16]. In recent years, most of the UAs have been light-cured, whereas chemical-cured adhesives are less common. Nevertheless, the disadvantages of light-activated adhesives are that they require a long irradiation time, and the irradiation energy has difficulty penetrating deep cavities sufficiently, resulting in poor polymerization [17].

Recently, a new universal chemically activated adhesive, Bondmer Lightless (Tokuyama Dental, Ibaraki, Japan), has been developed and commercialized around the world with the concept of “no wait” and “quick bonding,” characterized by the elimination of the light activation step to reduce the operation time [18]. According to the previous study, a novel acidic three-dimensional self-reinforcing monomer (3D-SR) has been developed and incorporated in Bondmer lightless, and it helps to maintain both the integrity and a uniform, thin, and strong bonding layer, thus ensuring the effective adhesion to teeth [19,20].

For the laboratory experiments, extracted human teeth are essential to evaluate the various properties of adhesives. According to the Academy of Dental Materials guidelines, a 0.5% chloramine-T solution has been commonly recommended as a medium to store the teeth after extraction [21,22]. Chloramine-T is an oxidizing agent but degrades easily in solutions, releasing hypochlorite ions (OCl^-^) [23]. It has been shown that storage in the chloramine-T solution for 2 years did not affect the bond strengths between dentin and the composite resin [24], although a previous report demonstrated that the short-term exposure to the chloramine-T solution induced increased surface porosity in the resin composites [25], and Camps et al. reported that it might increase the microleakage [26]. However, it is still unknown whether the short-term exposure of the chloramine-T solution affects dental adhesives’ bonding performance and mechanical properties.

Therefore, the objective of this research was to study the short-term exposure of a light-cured adhesive and a chemical-cured adhesive in the chloramine-T solution, to determine the effects on the dentin bonding performance and mechanical properties, while examining the resin–dentin interfacial characteristics with a focused ion beam/scanning ion microscope (FIB/SIM). The null hypotheses for this study were (i) the μTBS of both adhesive systems would not be influenced by the immersion solutions or the durations of immersion, and (ii) the mechanical properties and interfacial characteristics of the resin–dentin interface with different bonding agents would not be observed to vary when examined using the FIB/SIM technique.

## 2. Materials and Methods

An aqueous solution of 0.5% chloramine-T was prepared by dissolving 0.5 g of chloramine-T powder (Wako Pure Chemical Industry, Osaka, Japan) in 100 mL of distilled water with thoroughly mixing. This study used a total collection of 168 intact human molars without cavities [21], and approval was granted by the Ethics Committee of the Hokkaido University Faculty of Dentistry (#2018-09). 

The adhesives employed in the study are shown in Table 1.

### 2.1. Experimental Design and Bonding Procedure

**Figure 1:**
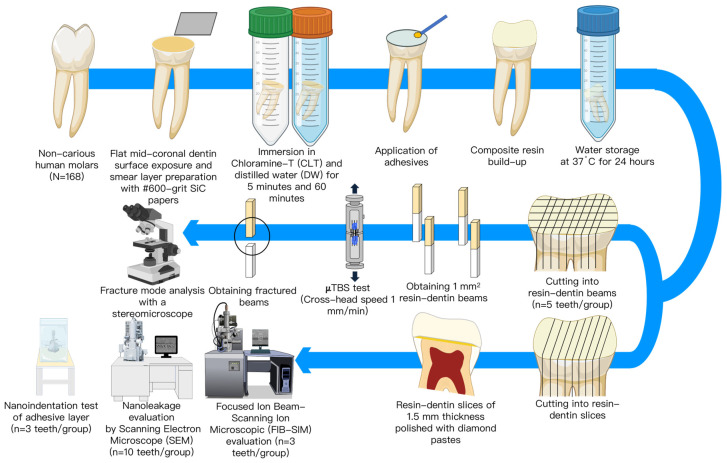
Schematic representation of the methodology.

This study used an in vitro approach (Figure 1). The 168 teeth were randomly assigned to eight groups based on the adhesive systems used [(Bond Force II (BF) and Bondmer Lightless (BL)]; the solutions used for immersion before bonding were 0.5% chloramine-T solution (CLT) and distilled water (DW); and the immersion durations were 5 and 60 min. To create standardized smear layers, flat mid-coronal dentin surfaces were exposed using a model trimmer (MT-7, J. Morita. CORP, Tokyo, Japan) under water cooling, and polishing for 1 min with 600-grit SiC paper (Fuji Star Type DDC, Sankyo Rikagaku Co. Ltd., Saitama, Japan). For the DW groups, the bonding process occurred after the polished dentin surfaces had been immersed in DW for either 5 or 60 min. For the CLT groups, the teeth were immersed in CLT for either 5 or 60 min prior to bonding. The samples were then taken out of the DW or CLT, and the dentin surface was dried. The two adhesives were applied according to the manufacturers’ instructions. Then, a layer of resin composite (Clearfil AP-X, shade A2, Lot. 170130, Kuraray Noritake Dental, Niigata, Japan) was built up to a thickness of 4 mm. The bonded teeth were then stored in distilled water at 37 °C for 24 h.

### 2.2. μTBS

A total of 40 teeth (*n* = 5) from the total 168 were used for μTBS. After water storage, the bonded teeth were cut into 1 mm^2^ cross-sections, then cut perpendicular to the bonded surfaces with a low-speed diamond saw (Isomet, Buhler, Lake Bluff, IL, USA). Then, these beams were fixed to a Ciucchi’s jig using cyanoacrylate glue (Model Repair 2 Blue, Dentsply Sirona, Tokyo, Japan) and subjected to tensile stress at a crosshead speed of 1 mm/min until failure, using a universal testing machine (EZ Test, Shimazu, Kyoto, Japan). The load (N) at the time of failure was recorded and divided by the bonded area to calculate the μTBS value in MPa. The mean μTBS of each tooth was considered as a statistical unit.

### 2.3. Fracture Mode Analysis and SEM Observation

Following the μTBS test, an optical microscope with a digital camera system (Moticam 1080, Shimazu) was used to inspect both sides of the fractured beams at 100× magnification. The fracture patterns were classified into four categories: A for adhesive failure, CC for cohesive failure in the resin composite, CD for cohesive failure in dentin, and M for mixed failure (extending into the dentin or resin composite).

Additionally, representative fractured beams from each group were selected and further analyzed using a field emission SEM (S-4800, Hitachi, Tokyo, Japan). The specimens were prepared for analysis with a layer of Pt-Pd and sputter-coated for 120 s. Finally, the SEM observations were performed at magnifications of 80× and 2000× with an accelerating voltage of 10 kV.

### 2.4. Nanoleakage Evaluation

Eighty teeth (*n* = 10) were used for the nanoleakage evaluation. After water storage at 37 °C for 24 h, the bonded teeth were sectioned, perpendicular to the adhesive–dentin interface to obtain slices with a thickness of 1.5 mm from each group. Two layers of fast-drying nail varnish were applied to the composite and dentin surface of the slices, except for a 1 mm width around either side of the adhesive layer. The slices were then immersed in a 50% ammoniacal silver nitrate solution (pH = 9.5) and prepared following the same protocol as Tay et al. [27] in darkness for 24 h.

Afterward, the specimens were rinsed thoroughly with running water, then immersed in a photo-developing solution for 8 h under fluorescent light, to reduce the silver ions to metallic silver grains. Following the removal from the developing solution, the specimens were thoroughly washed. The slices were embedded in epoxy resin (EpoFix Kit, Struers, Ballerup, Denmark), and the adhesive–dentin surfaces were polished sequentially with 600-, 800-, 1000-, and 1200-grit SiC papers under running water. The surfaces were then wet-polished sequentially with 6-, 3-, 1-, and 0.25-μm diamond pastes (DP-Paste, Struers) and ultrasonically cleaned. Following the air-drying, the specimens were sputter-coated with Pt-Pd for 120 s, and the adhesive–dentin interface was examined under the SEM using the backscattered electron mode at 10 kV. Images were obtained at 5000× magnification.

### 2.5. Nanoindentation Tests-Hardness (H) and Elastic Modulus (E) Measurements

For the nanoindentation measurement, 24 teeth (*n* = 3) were prepared in the same manner as mentioned above and sectioned into twenty-four resin–dentin slices (1 slice/tooth) after water storage (37 °C for 24 h). The samples were embedded in epoxy resin, with the resin–dentin interface facing outward, and polished using both the SiC papers and diamond pastes until the particle sizes were down to 0.25 μm. After being thoroughly dried, the nanoindentation test was conducted at 28 °C using a Berkovich indenter (ENT-1100a, Elionix, Tokyo, Japan) with a maximum load of 1 mN. The nanoindentation process consisted of three parts: 10 s for loading to the peak value, 1 s for holding at the peak load, and 10 s for unloading. Indentations were made along the middle of the bonding layer, with the indentation points set at the approximate half-width of the bonding layer and each following the indentation measured at intervals of at least 10 μm. The mean hardness and elastic modulus values of the three samples were determined for each group.

### 2.6. Cross-Sectional Focus Ion Beam/Scanning Ion Microscopy (FIB/SIM) Analysis

To assess the impact of FIB milling on the structural integrity of the adhesive–dentin interface, the resin–dentin slices were obtained in the same manner as mentioned above and embedded in epoxy resin. The adhesive–dentin surfaces were mechanically exposed, polished, dried, and then coated according to the previously described specimens. These samples were analyzed using an FIB/SIM system (JEM-9320FIB, JEOL Ltd., Tokyo, Japan) equipped with a gallium ion source, operated at 30 keV acceleration energy with 5000 pA ions currently at room temperature.

For each slice, a region of interest (ROI) measuring 20 μm × 30 μm was selected, including the resin–dentin interface. Prior to milling, a protective film of carbon was coated on the edge-plane of the ROI area to prevent the topmost surface from being affected by any potential damage or milling artifacts. The cross-section milling was performed from the edge-plane towards the ROI area using a dose of 5 nC/μm^2^ of ions, leaving an exposed rectangular perpendicular to the beam path and parallel to the interface. After the milling, the subsurface morphology of the samples was examined.

### 2.7. Statistical Analysis

The normality and homogeneity of the bond strength, hardness, and elastic modulus data were analyzed using the Shapiro–Wilk and Levene’s Equality of Error Variances tests. For the bond strength analysis, “tooth” was considered the statistical unit, and the data were examined using three-way ANOVA followed by Tukey’s test. Failure modes were analyzed for statistically significant differences using the non-parametric Pearson chi-square test. The hardness and modulus of the elasticity data were analyzed using the Kruskal–Wallis test, followed by the Mann–Whitney test. The level of significance was set at 5% (α = 0.05). All statistical analyses were performed using SPSS 26.0 for Windows (SPSS, Chicago, IL, USA).

## 3. Results

### 3.1. μTBS Test

**Figure 2:**
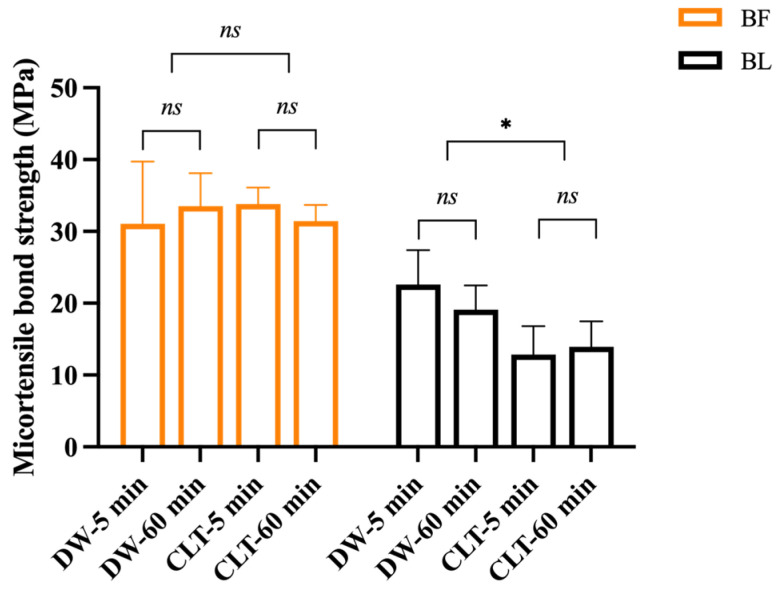
Bar graph representing the mean values and standard deviations of μTBS (MPa), (*n* = 5, * *p* < 0.05 indicates statistically significant difference; *ns* indicates no statistically significant difference). BF: Bond Force II; BL: Bondmer Lightless; DW-5 min: immersion in distilled water for 5 min; DW-60 min: immersion in distilled water for 60 min; CLT-5 min: immersion in 0.5% chloramine T solution for 5 min; CLT-60 min: immersion in 0.5% chloramine T solution for 60 min.

The results of the μTBS test are shown as a bar graph in Figure 2, displaying the mean ± SD values. Pre-testing failures were recorded in the BL groups as 0 MPa. A three-way ANOVA statistical analysis revealed a significant influence on the μTBS by the adhesives (*F* = 110.986, *p* < 0.001) and immersing solutions (*F* = 6.040, *p* < 0.05), but not by the immersing times (*F* = 0.158, *p* = 0.693). There was a statistically significant interaction between the adhesives and immersing solutions (*F* = 7.128, *p* < 0.05). Overall, the μTBS values were higher in the BF group compared with the adhesive BL group. The μTBS of the BL specimens immersed in CLT for 5 min showed significantly lower values than those immersed in DW for 5 min (*p* < 0.05). The μTBS values of the BL specimens immersed in DW for 60 min also tended to decrease compared with those immersed in CLT for 60 min; however, they were not noticeably different. Additionally, the immersing solutions and times did not have a significant influence on the μTBS of the BF group.

### 3.2. Fracture Mode Analysis and Representative SEM Images

Table 2 displays the percentage of failure modes for each group. A chi-square test revealed no significant differences in the failure mode distribution among the groups (*p* > 0.05). There were no cohesive failures in the resin composite observed in any of the testing groups. Cohesive failure in dentin was only observed in the BF group: 24% for DW-5 min, 13% for DW-60 min, 29% for CLT-5 min, and 9% for CLT-60 min. For the BF group, the predominant failure mode in both the DW and CLT groups was adhesive failure, although mixed failure and cohesive failure in dentin were also observed. In the BL specimens, the predominant failure mode in the DW groups was adhesive failure, followed by mixed failure. However, the percentage of adhesive failures in the CLT groups was higher than that in the DW groups (*p* > 0.05), increasing to 100%.

**Figure 3:**
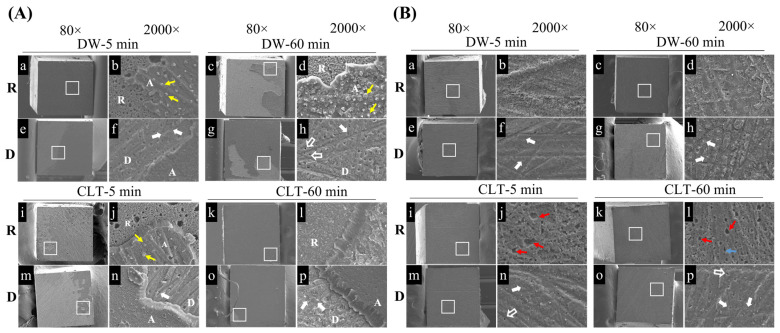
Representative SEM images showing adhesive fracture modes of the resin composite and dentin sides of BF and BL in each group. The selected areas (white square) in 80× images (**a**,**c**,**e**,**g**,**i**,**k**,**m**,**o**) of (**A**,**B**) are magnified to the 2000× images (**b**,**d**,**f**,**h**,**j**,**l**,**n**,**p**) of (**A**,**B**), respectively. R: resin composite; D: dentin; A: adhesive. In BF (**A**), partial adhesive remnants with resin tags (yellow arrowheads) could be seen on the surface of resin composite, while noticeable scratches and occluded dentinal tubules (white arrows) were seen on the corresponding dentin side. In BL (**B**), obvious scratches from surface preparation were observed on the dentin surfaces of each immersed sample (**f**,**h**,**n**,**p**). The openings of dentinal tubules (empty white arrows) and occluded dentinal tubules (white arrows) were shown on the surface of the dentin (**f**,**h**,**n**,**p**). The bubbles (red arrowheads) were found on the surface of the resin composite in the CLT-5 min and CLT-60 min groups (**j**,**l**).

Representative SEM images of the adhesive failures are shown in Figure 3. Images were taken from both the resin composite side (R) and dentin side (D) of the fractured beams in both the BF and BL materials. Figure 3(Aa,Ac,Ae,Ag,Ai,Ak,Am,Ao) and Figure 3(Ba,Bc,Be,Bg,Bi,Bk,Bm,Bo) show the overall views of the fracture surfaces on both the dentin and resin sides in the BF and BL materials. In the BF materials, the resin composite side revealed part of the adhesive attaching to the composite surface (Figure 3(Ab,Ad,Aj,Al)), while the polishing scratches and exposed dentin tubules could be seen in the images of the corresponding dentin side (Figure 3(Af,Ah,An,Ap)). This indicated that the adhesive failure occurred along both the adhesive–composite interface and the adhesive–dentin interface.

In the BL materials, numerous bubble structures were observed on the composite side of the CLT-exposed samples (Figure 3(Bj,Bl)), but not in the DW groups (Figure 3(Bb,Bd)). In addition, the adhesive fracture predominantly occurred along the adhesive–dentin interface with the presence of polishing scratches on the dentin surfaces (Figure 3(Bf,Bh,Bn,Bp)).

### 3.3. Nanoleakage Observation

**Figure 4:**
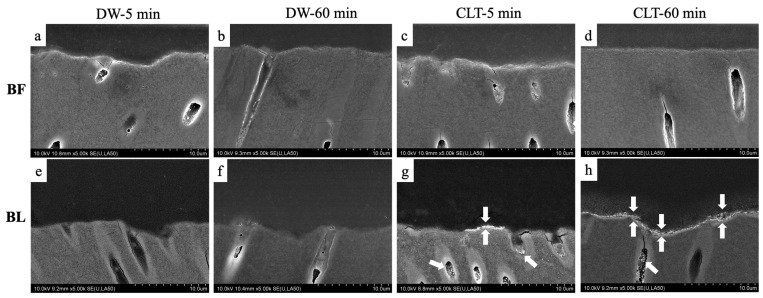
Backscattered SEM images of the interface bonded with BF (**a**–**d**) and BL (**e**–**h**) in different conditions magnified by 5000×. The silver deposits (white arrows) in a lateral tubule were indicated at the interface between the BL adhesive layer and dentin in the CLT-5 min and CLT-60 min conditions (**g**,**h**).

Figure 4 displays the representative SEM images of the adhesive–dentin interfaces under the backscattered mode. Nanoleakage was barely noticeable when immersed in DW. In the case of the BF groups, regardless of the immersing solutions or durations, only sparse silver deposits could be observed (Figure 4a–d).

In the BL groups, no obvious nanoleakage was seen after the sample immersion in DW (Figure 4e,f), whereas isolated silver grains were observed along the adhesive–dentin interfaces after the immersion in CLT. Following a 5 min immersion, silver was detected in dots (Figure 4g). By contrast, after 60 min of immersion, silver deposits appeared as a line along the adhesive–dentin interface and within the dentin tubules (Figure 4h).

### 3.4. Nanoindentation Tests

**Figure 5:**
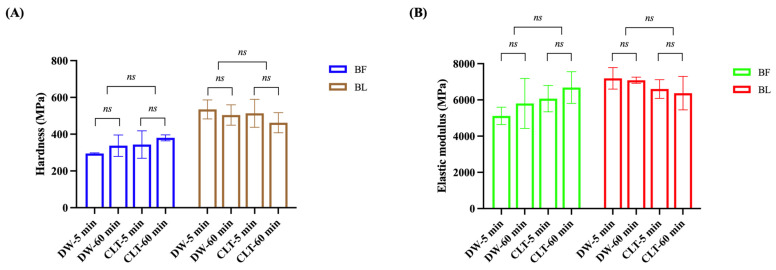
Bar graphs showing mean hardness (**A**) and elastic modulus (**B**) values (standard deviation) in MPa of the adhesive layer, respectively. *ns* indicates no statistically significant difference. BF: Bond Force II.

Figure 5 presents the values and standard deviations of the hardness and elastic modulus of the adhesive layers for each group. With respect to the hardness, Kruskal–Wallis tests revealed the overall values were generally higher in the BL groups than those of the BF groups. BF did not show statistically significant differences between the DW-5 min and DW-60 min groups or between the CLT-5 min and CLT-60 min groups in terms of hardness (*p* > 0.05), as shown in Figure 5A. Meanwhile, BL also showed no significant differences between the DW-5 min and DW-60 min groups (*p* > 0.05) or between the CLT-5 min and CLT-60 min groups in terms of hardness (*p* > 0.05), as shown in Figure 5A. For the elastic modulus, same as the hardness results, neither the immersing solution nor the immersing time showed any significant difference for the elastic modulus in the BF and BL groups (*p* > 0.05), as shown in Figure 5B.

### 3.5. Cross-Sectional FIB/SIM Observation

**Figure 6:**
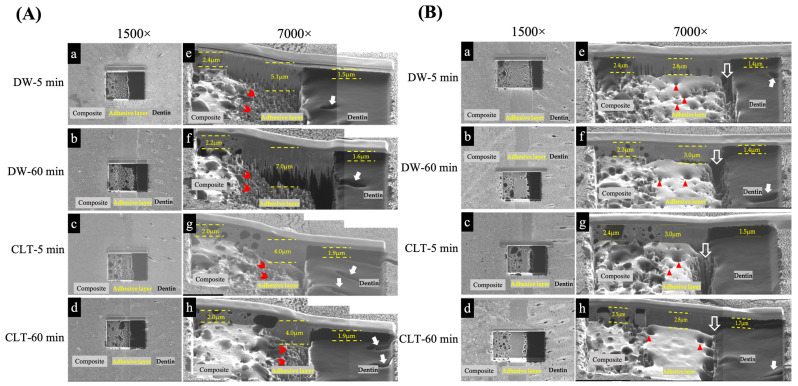
Interfacial morphologies of resin–adhesive–dentin after FIB milling of BF (**A**) and BL (**B**) in each group. Morphological investigation of the composite–adhesive–dentin interface was observed from the top view at 1500× magnification in each group (**a**–**d**). The tilted views (60°) of the sample surfaces were obtained at 7000× magnification (**e**–**h**). Filled white arrows showed the appearance of tubules on the dentin surface (**Ae**–**Ah**,**Be**–**Bh**). Empty white arrows in (**Be**–**Bh**) showed a ditch-like appearance between the BL adhesive layer and dentin structure. The red arrowhead showed cone-like projections in adhesive layer of BF (**Ae**–**Ah**), while red triangle indicated small voids in adhesive layer of BL (**Be**–**Bh**).

Figure 6 shows the morphological observations of BF and BL. The top views of the interfacial morphology after FIB milling at a magnification of 1500× are presented in Figure 6(Aa–Ad) and Figure 6(Ba–Bd). The sample surfaces were removed through sputtering, resulting in a rectangular box with a non-uniform bottom surface. In the composite area of all groups, redeposited structures with a melted appearance were observed, while dentin appeared as a homogeneous black zone. The adhesive layer in the BL group was relatively flat and smooth in contrast with the BF group, where spots created by sputtering were evident, particularly near the interface between the adhesive and dentin.

Upon tilting the samples by 60° and imaging them at 7000× magnification, a tilted view of the specimens was obtained in Figure 6(Ae–Ah) and Figure 6(Be–Bh). The morphological features of the resin and dentin regions were consistent with each group, where the resin composite exhibited non-uniform and melted characteristics, while the dentin surface displayed regular and homogeneous features, with visible dentinal tubules. The milling of the different surfaces (composite, adhesive, and dentin) resulted in varying depths for each group. The overall milled depth of the adhesive layer in the BF groups was greater than that of the BL groups. Furthermore, the FIB milled adhesive layer of BF exhibited a unique appearance with cone-like projections (Figure 6(Ae–Ah)), whereas the BL group displayed smooth and regular surfaces with several small spots (Figure 6(Be–Bh)). Notably, the depth of milling in the BF group was observed to be dependent on the immersing solution, with the CLT groups (Figure 6(Ag,Ah)) producing a shallower adhesive layer than the DW groups (Figure 6(Ae,Af)). Moreover, a ditch-like appearance with a 1–2 mm width was observed between the BL adhesive layer and the dentin structure (Figure 6(Be–Bh)).

## 4. Discussion

The human teeth used for research and teaching purposes are a potential source, especially in the in vitro bonding studies of enamel or dentin [28]. To maintain the fresh condition of teeth after extraction, chloramine T (CLT) is commonly used as a storage solution in adhesion studies; however, the duration varies among studies, ranging from a few hours to several months [29]. Previous study indicated that the bond strength of dentin tended to decline after 2 years of storage with chloramine T [24]. In contrast, Retief et al. demonstrated a tendency for the shear bond strength of dentin to increase with prolonged storage [30].

In the current investigation, the results from a three-way ANOVA revealed that the adhesives and immersing solutions significantly influenced the μTBS (*p* < 0.001 and *p* < 0.05, respectively), allowing the rejection of the first null hypothesis. Particularly, the immersion in the CLT solution significantly reduced the bonding efficacy of BL to dentin, possibly as a result of the inhibitory effects of residual CLT in the dentinal tubule on the chemical reaction of adhesive BL. This inhibition may have influenced the polymerization of the adhesive interface, leading to a significant reduction in μTBS [31]. Conversely, the μTBS of BF was not reduced in different immersing solutions, possibly due to the use of camphorquinone as a photoinitiator, which caused rapid hardening and the high levels of polymerization of TEGMA and Bis-GMA without being adversely affected by CLT [32].

Since the microtensile bond strength test was developed to evaluate the bond strength between the bonding materials and a small area of dental tissue, which should theoretically produce a more uniform stress distribution at the interface, the fracture was expected to occur in the adhesive failure mode predominantly [33]. The current study showed the same results, with the dominant fracture mode for both materials being adhesive failure. According to previous researchers, they believed that the failure mode is related to the bond strength, with the percentage of adhesive failure decreasing as the bond strength of one-step adhesive increased [34]. This is consistent with the results of this study, that the BF groups with higher bond strengths showed lower percentages of adhesive failure compared with the BL groups. Small bubbles were found at the fracture surfaces of the BL bonding resin (Figure 3(Bj,Bl)), and this induced an unfavorable bonding quality. It may be that the solvents (acetone, isopropyl alcohol, and water) cannot be easily removed by blowing air, resulting in the insufficient polymerization of the resin monomers, leaving the solvents to remain in the adhesive resin layer. The same bubbles and voids were also found in the previous study [35]. In general, cohesive failure often occurs on the specimens with high bond strengths [36]. But, one literature demonstrated that there was no correlation between them [37]. Therefore, further studies are required to determine the relationship between the bond strength and fracture modes.

A new three-dimensional self-reinforcing (3D-SR) adhesive monomer was developed and incorporated in both the BF and BL materials and has the potential to chemically bond to the tooth structure by forming multiple bonding sites with calcium. Additionally, the phosphate group of the 3D-SR adhesive monomer can form ionic bonds with the free calcium ions present at the adhesive interface during dentin demineralization [20,38]. The overall μTBS value of BF was higher than that of BL (Figure 2), potentially due to the significantly higher abundance of 3D-SR monomers in BF (10–30%) compared with BL (1–5%) [39,40,41], which might contribute to more chemical reactions between dentin and BF to achieve a relatively better bonding efficacy. Moreover, some studies showed that chemical-cured cements exhibited significantly lower bond strengths than light-cured cements [42,43]. The outstanding advantage of light-cured materials is that the polymerization does not occur until light irradiation; therefore, the operation time can be adjusted freely. The polymerization of chemical-cured cements requires a polymerization initiator, but the polymerization speed is slow due to the constant consumption of the initiator [32]. The polymerization of dual-cured cements under the chemical-cured mode is much slower than that under the light-cured mode. This may also explain why the bonding performance of chemical-cured materials is not as effective as light-curing materials [44,45].

A nanoleakage evaluation technique using silver nitrate staining provides an indirect method to evaluate the quality of resin–dentin bonds and to identify the potential defects or nano-sized voids within the hybrid layer and the adhesive layer [46,47]. The hydrophilic mono-functional monomer 2-hydroxyethyl methacrylate (HEMA) is often added into adhesives to promote monomer diffusion into the demineralized substrate [48]. In this study, the expression of nanoleakage differed between the two adhesives. We found traces of silver within the resin–dentin interface only in the BL group, mainly after the CLT immersion, which could be explained by the higher concentrations of HEMA (10–30%) in the BL adhesive [39,40,41]. According to reports in the literature, in one-step, self-etch adhesive systems, higher concentrations of HEMA enhance the osmosis of water through the adhesive layer, resulting in numerous droplets coming into contact with dentin [48]. BL is a two-bottle, one-step, acetone/water-based adhesive system. The manufacturer’s instructions suggest using strong air blow during usage, to evaporate the water from the acetone-based adhesives. This may help reduce or eliminate the phase separation, by causing the removal of residual water. However, residual water in acetone-based adhesives cannot be easily and completely removed [49]. All these reasons might provide more pathways for silver penetration, thereby increasing the possibility of nanoleakage. Another possible reason is that the CLT has a strong oxidation activity [50], which might lead to the insufficient chemical polymerization of BL at the interface. This insufficient polymerization could create a weak zone in the resin–dentin interaction, allowing more water to remain at the interface [51].

In this study, no significant differences were found in the hardness and elastic modulus between the different time frames and immersion solutions in both the BF and BL groups (Figure 5). However, the overall hardness value was higher in BL than BF, which might be because of the presence of different concentrations of Bis-GMA in the BL (10–30%) and BF (5–15%) concentrations [39,40,41]. Bis-GMA is a hydrophobic dimethacrylate with a high molecular weight, low polymerization shrinkage, and fast hardening ability, making it commonly used in dental adhesives and composite resins [52]. It can provide mechanical strength by forming dense crosslinking polymers in the adhesive systems [53,54]. We speculated that this higher content of Bis-GMA in BL can support a better crosslinking network [55], contributing to the improved mechanical properties over BF. Although BF had relatively lower hardness and elastic modulus values, it still gave adequate adhesive resistance to the elastic deformation under stress, maintaining a high bond strength [55]. This result is consistent with a study by Freitas et al. showing that a lower elastic modulus values yielded higher μTBS [56]. Van Meerbeek et al. demonstrated that, rather than the adhesive layers, the resin–dentin transition contributed more to the bond strength by relieving the stresses between the shrinking composite resin and the rigid dentin [57].

In searching for a novel technique to determine the interaction quality and inner morphological characteristics of the resin–dentin interface, this study evaluated the usefulness of FIB/SIM. This technique integrates processing and imaging technologies into a single instrument, enabling both milling and observation [58]. It operates in a similar manner to SEM, with the main difference being ions, rather than electrons, interacting with the surface to generate secondary electron signals, yielding an improved contrast [59]. The FIB/SIM analysis showed different interfacial characteristics of the resin–dentin interface between the BF and BL materials, allowing the second null hypothesis in this study to be rejected. Following the FIB milling, the subsurface structures were exposed, revealing the details of underlying morphologies between the materials. In BL and BF, FIB milling revealed a transition zone between the adhesive and dentin (Figure 6). Young et al. described that the ion beams preferentially milled “softer” materials [60], and our study used interfacial FIB-SIM imaging to confirm that the milling depth varied in different media (composite, adhesive, and dentin). Further BF adhesive layers were milled via ion beams, indicating that the milled depth of BF (Figure 6(Ae–Ah)) was greater than that of BL (Figure 6(Be–Bh)). The lower hardness of the BF adhesive probably resulted in a vulnerable area, which could be easily milled via FIB. However, the ion beam also created a cone-like structure on the BF adhesive layer, and further research is required to investigate the reason for this. Ditch-like features were observed in the BL groups, which were possibly caused by most of the bonding fractures occurring at the interface between the adhesive layer and dentin. Moreover, this ditch-like appearance was only found in between the BL adhesive layer and the dentin structure, suggesting that the BL adhesive interface might not have formed a strong bond with the dentin. This is possibly caused by the incomplete polymerization of the BL adhesive at the adhesive–dentin interface. Moosavi et al. have speculated that chemical-cured specimens can result from a slow polymerization rate and a weak development of cross-links by curing [61]. The incomplete polymerization of the adhesive, forming an unstable adhesive layer, may cause an increased permeability of the bonded interfaces [62]. This allows more water to penetrate from the underlying dentin to the adhesive layer, and the water may be trapped by a slow-curing material, compromising the quality of the adhesive–dentin interface [63].

This study, as it must be noted, is not free from limitations. Only qualitative analyses were evaluated for the nanoleakage and focus ion beam/scanning ion microscopy at the resin–dentin interface. The correlation between the microtensile bond strength and silver penetration at the resin–dentin interface still needs to be determined. In addition, we recommend further research regarding the quantitative analysis of the relationship between the FIB milling depth and the mechanical properties of the adhesives.

Nonetheless, it is very important to evaluate the clinical performance of dental adhesives in a more clinically relevant environment [64]. Also, considering the direct contact of dental adhesives with both hard and soft dental tissues, the evaluation of biocompatibility, bioactivity, and biodegradability of dental adhesives needs to be addressed critically [65,66]. To the best of our knowledge, no study has been conducted to evaluate the cytotoxic and other biological effects of these two dental adhesives. Our future endeavors should include both in vitro and in vivo investigations to assess the biological and clinical aspects of these materials via evaluation using cytotoxicity and genotoxicity assessments, apoptosis detection assays, and an examination of the cell cycle progression. Further, in order to improve the bond strength of these materials, the application of ion-gel and hydrogel in a clinically mimicked environment need to be determined as previously described [67,68].

## 5. Conclusions

The bond strengths of the light-cured adhesive, Bond Force II, were higher than those of chemical-cured adhesive, Bondmer Lightless. The bonding performance of the chemical-cured adhesive is compromised by the short-term immersion of the chloramine-T solution, as evidenced by the observed decrease in bond strength and increase in nanoleakage. However, in the adhesives studied, the mechanical properties and morphological characteristics of the resin–dentin interfaces remain unaffected by the solutions. And, in terms of the materials used, the lower hardness of Bond Force II makes it more susceptible to ion-beam milling. The newly developed FIB/SIM system can accurately assess the three-dimensional morphological characteristics of the resin–dentin interactions and indirectly reflect the mechanical properties of the adhesive systems by measuring the milling depth, but standards and protocols still need to be established.

## Figures and Tables

**Table 1:** Adhesives used in this study.

Adhesives	Compositions	Manufacturer’s Instructions
Bond Force II (pH = 2.8) (Tokuyama Dental, Ibaraki, Japan, Lot. 036098)	Phosphoric acid monomer (3D-SR monomer), Bis-GMA, TEGDMA, CQ, HEMA, alcohol, water.	1. Apply adhesive to the dentin surface using a micro-brush and leave for 10 s. 2. Apply mild air for approximately 5 s. 3. Light cure for 10 s.
Bondmer Lightless (pH = 2.2) (Tokuyama Dental, Ibaraki, Japan, Lot. 0430Y8)	Liquid A: acetone, Phosphoric acid monomer (3D-SR monomer), Bis-GMA, TEGDMA, HEMA, MTU-6, and others. Liquid B: acetone, isopropyl alcohol, water, borate catalyst, γ-MPTES, peroxide, and others.	1. Take a drop of liquid A and a drop of liquid B and mix them evenly. 2. Apply adhesive to the dentin surface using a micro-brush (within 30 s). 3. Gently air blow until the liquid surface stops moving, then stronger air blow to completely dry.

3D-SR monomer: three-dimensional self-reinforcing monomer; Bis-GMA: 2,2-bis [4-(2-hydroxy-3-methacryloyloxypropoxy) phenyl)] propane; TEGDMA: triethyleneglycol dimethacrylate; HEMA: 2-hydroxyethyl methacrylate; MTU-6: 6-methacryloyloxyhexyl-2-thiouracil-5-carboxylate; γ-MPTES: c-methacryloyloxypropyltriethoxysilane; CQ: dl-camphorquinone.

**Table 2:** The percentage of fracture modes (A/M/CD/CC).

		A	M	CD	CC
BF	DW-5 min	69%	7%	24%	0%
	DW-60 min	76%	11%	13%	0%
	CLT-5 min	54%	17%	29%	0%
	CLT-60 min	81%	11%	9%	0%
BL	DW-5 min	92%	8%	0%	0%
	DW-60 min	86%	14%	0%	0%
	CLT-5 min	100%	0%	0%	0%
	CLT-60 min	100%	0%	0%	0%

A, adhesive failure; M, mixed failure; CD, cohesive failure in dentin; CC, cohesive failure in composite resin.

## Data Availability

The data presented in this study are available on request from the corresponding author.
